# Factors associated with anti-phenolic glycolipid-I seropositivity among the household contacts of leprosy cases

**DOI:** 10.1186/s12879-015-0955-3

**Published:** 2015-05-30

**Authors:** Ana Paula Mendes Carvalho, Angélica da Conceição Oliveira Coelho Fabri, Rodrigo Corrêa Oliveira, Francisco Carlos Félix Lana

**Affiliations:** Postgraduate Program in Nursing, Escola de Enfermagem, Universidade Federal de Minas Gerais−UFMG, Belo Horizonte, MG Brasil; Department of Basic Nursing, Faculdade de Enfermagem, Universidade Federal de Juiz de Fora−UFJF, Juiz de Fora, MG Brasil; Laboratory of Cellular and Molecular Immunology, Centro de Pesquisas René Rachou−CPqRR, Fundação Oswaldo Cruz−FIOCRUZ, Belo Horizonte, MG Brasil; Laboratory of Immunology, Núcleo de Pesquisas em Ciências Biológicas, Universidade Federal de Ouro Preto−UFOP, Ouro Preto, MG Brasil; Instituto Nacional de Ciência e Tecnologia em Doenças Tropicais−INCT-DT, Belo Horizonte, Brasil; Department of Maternal and Child Nursing and Public Health, Escola de Enfermagem, Universidade Federal de Minas Gerais−UFMG, Belo Horizonte, MG Brasil

## Abstract

**Background:**

The diagnosis of leprosy is based on clinical symptoms of the disease, which may not be sufficient to ensure early diagnosis. The development of effective tools for the early detection of infection, such as rapid tests that can be applied by non-specialists for early-stage leprosy identification, has been considered a research priority and may contribute to overcoming the complications associated with late diagnosis. The aim of this study was to analyze the factors associated with anti-phenolic glycolipid-I (PGL-I) seropositivity among the household contacts of leprosy cases.

**Methods:**

A cross-sectional study of individuals from the northeastern municipalities of the state of Minas Gerais, Brazil, was performed. Anti-PGL-I seropositivity was evaluated by assessing specific antibody production using the ML Flow test. A Poisson regression with a robust error variance was used to evaluate the relationship between anti-PGL-I seropositivity and the independent variables investigated.

**Results:**

The overall anti-PGL-I seropositivity was 13.5 %, and among the contacts of leprosy cases that were classified as paucibacillary or multibacillary, it was 8.4 and 17.3 %, respectively. The factors associated with the variation of anti-PGL-I seropositivity among the study population were the presence of signs suggestive of leprosy (PR = 3.68; 95 % CI: 1.56–8.71), the operational leprosy classification (PR = 2.17; 95 % CI: 1.22–3.86) and grade 1 (PR = 1.83; 95 % CI: 1.02–3.26) or grade 2 disability (PR = 2.42; 95 % CI: 1.02–5.47) of the index leprosy case.

**Conclusions:**

The presence of signs suggestive of leprosy and the operational classification of leprosy cases were associated with anti-PGL-I seropositivity. The serological tests available for leprosy are not considered to be diagnostic tests but can be used as auxiliary assessments in combination with clinical parameters to identify exposed individuals at high risk of developing leprosy and those exhibiting the initial stages of this disease.

## Background

The diagnosis of leprosy is essentially clinical [1, 2] and is made via an analysis of the history and living conditions of the patient and dermatologic examination [2, 3]. The slit skin smear is among the most widely used supplementary assessments to confirm this diagnosis and to determine the operational classification of leprosy. Other assessments, such as histopathological examination, the Mitsuda reaction, serological tests and the identification of *Mycobacterium leprae* via polymerase chain reaction (PCR) are also used [1].

Serological tests that identify the production of antibodies against phenolic glycolipid-I (PGL-I), a specific *M. leprae* antigen [4], have been used for patient classification, treatment monitoring, risk assessment for relapse and the selection of household contacts who exhibit an elevated risk of becoming ill [5, 6]. Individuals who live with leprosy patients have an increased risk of developing the disease compared to the general population [7].

The use of alternative tests associated with periodic clinical assessment can help to identify any household contacts infected and new cases of leprosy [8], particularly because dermatological examination may not be sufficient to ensure early diagnosis, as it is based on the manifestation of characteristic signs and symptoms of the disease.

The development of effective tools for the early detection of infection [9], such as rapid tests that can be applied by non-specialists for the identification of leprosy at an early (subclinical) stage, have been considered a research priority [10] and may contribute to overcoming the complications associated with late diagnosis.

Serological screening and follow-up of household contacts of leprosy patients, who exhibit the greatest risk of developing this disease, may represent a useful strategy for leprosy control and the early detection of new cases. The aim of this study was to analyze the factors associated with anti-PGL-I seropositivity among the household contacts of leprosy cases.

## Methods

This cross-sectional study was performed in the northeastern municipalities of the state of Minas Gerais, Brazil, located in the microregion of Almenara, which is composed of 16 municipalities. The municipal seat, Almenara, and an additional 13 municipalities are components of cluster 6, a region in Brazil known to contain a high concentration of leprosy cases [11].

The selected municipalities had reported the largest number of leprosy cases and household contacts in the Notifiable Diseases Information System (SINAN) from 2006 to 2010. Seven municipalities were selected for examination: Almenara, Felisburgo, Jacinto, Jordan, Palmópolis, Rubim and Santa Maria do Salto.

The population base of the study included the household contacts of leprosy cases reported in SINAN during the period of 2006–2010, as well as those cases diagnosed during data collection (from January to July 2011) by local health services. The household contacts included every individual residing in the same house or terrain as the index leprosy case at diagnosis. The inclusion criteria for the household contacts were 7 years of age or greater, having resided with the index leprosy case at the time of diagnosis and no personal history of leprosy.

The dependent variable was the serological status of the household contacts, based on the production of anti-PGL-I according to the rapid lateral flow test for *Mycobacterium leprae* (ML Flow), which is an immunoassay composed of one side of a nitrocellulose strip containing detection reagent and anti-human immunoglobulin M (IgM) and the other side containing an absorbent strip. For the test line, a semi-synthetic PGL-I antigen bound to bovine serum albumin (BSA) is used. In a second line parallel to the antigen line, the human IgM antibody serves as a control reagent. The complex is stored in a plastic device with a receptacle for the application of the sample to the absorbent paper and a window with an indicator for the control line and test reading. For the test, 5 μl of serum or undiluted whole blood and 130 μl of buffer solution are added. The reading is performed after 10 or 5 minutes when using serum or whole blood, respectively, and the result is considered valid only when the control line is clearly visible. The result is considered positive when distinct color in the test line is observed and is considered negative when no staining or weak staining is observed [5].

The independent variables included socio-demographic characteristics, living conditions, the cohabitation of the household contact with the index leprosy case, Bacillus Calmette-Guérin (BCG) vaccination status, the presence of signs suggestive of leprosy and the clinical characteristics of the index leprosy case.

The SINAN database and patient charts were used to identify the index leprosy cases and the related clinical information. Data collection was conducted via home visits, preceding contact with a local health professional. During the home visits, all household contacts received a dermatological evaluation to identify any signs or symptoms suggestive of leprosy, which were determined by the presence of at least one of the following alterations: spots; spots with abnormal sensitivity, assessed using an esthesiometer; plates; tubers; nodules; infiltration; neural thickening of the ulnar, radial, radial cutaneous, fibular and posterior tibial nerves; reduced muscle strength in the eyelid and abductor muscles of the fifth finger, thumb abductor, the wrist extensor, extensor valgus, long extensor digitorum, anterior tibial and fibular muscles; and sensitivity changes in the eyes, hands and feet.

The contacts who exhibited characteristic signs of leprosy were referred to health services to confirm the diagnosis. The clinical, epidemiological and demographic data were obtained via completion of a structured questionnaire, and a drop of blood was collected via finger prick for the ML Flow test.

The participants were informed during data collection of the implications of the ML Flow test and the signs, symptoms and clinical manifestations of the different forms of leprosy and were advised to seek health services if any characteristic signs of the disease were observed.

The Epi Info software was used to compile the database and the questionnaires, which were entered in duplicate to ensure the reliability of the information. Statistical Package for Social Sciences (SPSS) software version 18.0 and Statistical Software for Professionals (STATA), version 9.0 were used for statistical analysis.

The clinical characteristics of the leprosy cases identified during the 2006–2010 period who could not be located were compared to the characteristics of those who were located, and the Chi-square test was used to assess whether these losses were systematic. The prevalence ratio (PR) was calculated using a Poisson regression with a robust error variance to analyze the association between anti-PGL-I seropositivity and various factors, as the study design was cross-sectional and the outcome was frequent [12].

The criteria used for the inclusion of variables in the multivariate analysis model were p ≤ 0.20 based on the bivariate analysis and epidemiological plausibility. The variables were entered individually into the regression model, and those that lacked significance were excluded. The level of statistical significance was 5 % (*p* ≤ 0.05) for the multivariate analysis, and the Hosmer & Lemeshow test was used to validate the ultimate robustness of the model.

This study is a component of the research “Transmission and control of leprosy in the microregion of Almenara−Minas Gerais: relations between the organization of health services and people’s knowledge about the disease,” which was approved by the Ethics Committee in Research of the Universidade Federal de Minas Gerais and met the provisions established by the Declaration of Helsinki. All participants signed an informed consent form and authorized the collection of the sample for the ML Flow test. The informed consent form for children under 18 years of age was signed by the participant and by either a parent or a legal guardian.

## Results

The study participants consisted of 393 household contacts, 53 of whom displayed a positive result on the ML Flow test, corresponding to a seropositivity of 13.5 %. Table 1 presents the analysis of anti-PGL-I seropositivity in relation to the socio-demographic characteristics, BCG vaccination status and the presence of signs suggestive of leprosy.

**Table 1:** Association of socio-demographic characteristics, BCG and signs suggestive of leprosy with seropositivity among household contacts

Variable	Total of participants	Anti PGL-I seropositivity	PR	95 % CI	*P* value
	n	n (%)			
Gender					
Male	169	19 (11.2)	1		
Female	224	34 (15.2)	1.35	0.80–2.28	0.262
Age (years)					
7 to 14	102	19 (18.6)	1		
≥15	291	34 (11.7)	0.63	0.37–1.06	0.815
Educational level (years of study)					
<01	67	8 (11.9)	1		
01 to 08	245	36 (14.7)	1.23	0.60–2.52	0.571
09 to 11	70	7 (10.0)	0.84	0.32–2.16	0.714
≥12	11	2 (18.2)	1.52	0.36–6.48	0.570
BCG vaccination status					
No scar	82	8 (9.8)	1		
One scar	184	22 (12.0)	1.23	0.58–2.61	0.598
Two scars	127	23 (18.1)	1.86	0.88–3.93	0.107
Signs suggestive of leprosy					
No	381	47 (12.3)	1		
Yes	11	5 (45.5)	3.68	1.56–8.71	0.003

*PR* prevalence ratio, *95 % CI* 95 % confidence interval

The household contacts who exhibited signs suggestive of leprosy displayed a significantly higher anti-PGL-I seropositivity than those who exhibited no characteristic signs of the disease (PR = 3.68, *p* = 0.003). Furthermore, it is important to emphasize the high proportion of household contacts with signs suggestive of leprosy among individuals who showed anti-PGL-I-positive serology (9.62 %) compared to contacts showing signs suggestive of leprosy in the ML Flow test-negative group (1.76 %).

Anti-PGL-I seropositivity was also higher among female contacts (PR = 1.5), those aged 7 to 14 years (PR = 1), individuals who had 1–8 years of education (PR = 1.23) and 12 or more years of education (PR = 1.52) and the contacts who possessed one or two BCG scars (PR = 1.23 and PR = 1.86, respectively); however, these differences were not significant.

The analyses of anti-PGL-I seropositivity and the characteristics related to household contact cohabitation with the index leprosy cases (Table 2) demonstrated that seropositivity was elevated among contacts who lived in households with more than one occupant per bedroom (PR = 1.70). The seropositivity was higher for household contacts who shared the same bedroom with the leprosy case (PR = 1.31). Differences in seropositivity related to housing conditions were not significant.

**Table 2:** Association of consanguinity with the leprosy cases and housing conditions with seropositivity among household contacts

Variable	Total of participants	Anti PGL-I seropositivity	PR	95 % CI	*P* value
	n	n (%)			
Number of residents per room					
≤1	329	44 (13.4)	1		
>1	64	9 (14.1)	1.05	0.54–2.05	0.883
Number of residents per bedroom					
≤1	48	44 (91.7)	1		
>1	345	296 (85.8)	1.70	0.64–4.52	0.284
Cohabitation condition					
Sleep in the same terrain	28	3 (10.7)	1		
Sleep in the same household	228	31 (13.6)	1.27	0.42–3.84	0.673
Sleep in the same bedroom	128	18 (14.1)	1.31	0.42–4.11	0.641
Cohabitation status					
Resided with the leprosy case at the time of diagnosis	7	0 (0.0)	−	−	−
Resided with the case from diagnosis to present	383	52 (13.6)	−	−	−
Consanguinity with the leprosy cases					
No	120	17 (14.2)	1		
Yes	273	36 (13.2)	0.931	0.54–1.59	0.794

*PR* prevalence ratio, *95 % CI* 95 % confidence interval

Regarding the cohabitation conditions, no difference was observed in the prevalence of anti-PGL-I seropositivity with respect to the consanguinity of the contacts with the corresponding index leprosy case.

The analyses of the relationship between anti-PGL-I seropositivity and the clinical characteristics of the index leprosy cases are shown in Table 3.

**Table 3:** Association of the characteristics of the index leprosy cases with seropositivity among the household contacts

Variable	Total of participants	Anti PGL-I seropositivity	PR	95 % CI	*P* value
	n	n (%)			
Operational classification					
Paucibacillary	172	14 (8.1)	1		
Multibacillary	204	36 (17.6)	2.17	1.22–3.86	0.009
Number of lesions					
≤5	244	29 (11.9)	1		
>5	145	24 (16.6)	1.39	0.84–2.31	0.199
Clinical form					
Indeterminate	113	13 (11.5)	1		
Tuberculoid	73	3 (4.1)	0.36	0.11–1.16	0.087
Borderline	119	19 (16.0)	1.39	0.72–2.69	0.332
Virchownian	72	14 (19.4)	1.69	0.83–3.43	0.147
Slit skin smear status					
Negative	199	26 (13.1)	1		
Positive	103	13 (12.6)	0.97	0.52–1.81	0.914
Bacterial index					
<2	21	3 (14.3)	1		
≥2	361	47 (13.0)	0.91	0.31–2.71	0.868
Degree of disability at diagnosis					
Degree 0	215	19 (8.8)	1		
Degree 1	136	22 (16.2)	1.83	1.02–3.26	0.041
Degree 2	28	6 (21.4)	2.42	1.02–5.74	0.045
Number of damaged nerves					
≤1	70	5 (7.1)	1		
>1	69	11 (15.9)	2.23	0.82–6.08	0.119
Year of diagnosis					
2006	82	9 (11.0)	1		
2007	76	14 (18.4)	1.68	0.77–3.68	0.197
2008	79	10 (12.7)	1.15	0.50–2.68	0.741
2009	82	9 (11.0)	1	0.42–2.38	1.000
2010	47	5 (10.6)	0.97	0.35–2.70	0.952
2011	339	5 (25.0)	2.28	0.82–6.35	0.116

*PR* Prevalence ratio, *95 % CI* 95 % confidence interval

The seropositivity was analyzed according to the operational classification of the index leprosy case. The seropositivity was 8.4 % among the household contacts of paucibacillary cases and 17.3 % among the contacts of multibacillary leprosy cases. The household contacts of multibacillary leprosy cases displayed a significantly higher seropositivity than the contacts of paucibacillary cases (PR = 2.17; *p* = 0.009).

The anti-PGL-I seropositivity was lower for the contacts of tuberculoid cases than for the contacts of indeterminate cases (PR = 0.36). The seropositivity was higher among the contacts of other clinical forms of leprosy (borderline or virchownian) than among the contacts of the indeterminate form. A higher anti-PGL-I seropositivity was detected among the household contacts of patients with more than one affected nerve compared to the household contacts of patients with one or no affected nerves. These differences were not significant.

The seropositivity was not significantly different between the household contacts of leprosy cases with positive and negative results on the slit skin smear test or between those of leprosy cases with a bacterial index of greater than or equal to two and less than two.

The household contacts of cases with some degree of physical disability were increasingly seropositive compared to the contacts of cases diagnosed without any physical disability. The seropositivity was significantly higher among the contacts of patients with grade 2 disability than among the contacts of patients without any disability at the diagnosis (PR = 2.42; *p* = 0.045).

No significant differences were detected between the clinical characteristics of the leprosy cases who were located, whose contacts were included in the study, and the leprosy cases who were not located (Table 4).

**Table 4:** Comparasion of the clinical characteristics of the index leprosy cases located and not located

Variable	Leprosy cases located		Leprosy cases not located		*P* value*
	n	%	n	%	
Operational classification					
Paucibacillary	72	47,7	79	44,6	0,581
Multibacillary	79	52,3	98	55,4	
Clinical form					
Indeterminate	48	31,8	54	30,5	0,732
Tuberculoid	25	16,6	24	13,6	
Borderline	52	34,4	13	34,5	
Virchownian	26	17,2	38	21,5	
Slit skin smear status					
Negative	23	17,3	15	9,7	0,115
Positive	16	12,0	15	9,7	
Degree of disability at diagnosis					
Degree 0	81	54,0	99	55,9	0,555
Degree 1	58	38,7	70	39,5	
Degree 2	11	7,3	8	4,5	
Number of lesions					
≤ 5	112	74,2	120	68,6	0,226
> 5	39	25,8	55	31,4	

*Chi-square test. The comparison was not made for the variables number of damaged nerves and bacterial index because many leprosy cases do not have this information in the notification form or in the patient chart

*PR* Prevalence ratio, *95 % CI* 95 % confidence interval

Analysis of anti-PGL-I seropositivity by year of diagnosis revealed that the seropositivity was higher among the household contacts of patients who were diagnosed more recently, with 2006 as the base year, except for the contacts of cases diagnosed in 2009 and 2010. The highest seropositivity was observed among the contacts of cases diagnosed in 2011 (PR = 2.28). These observed differences were not significant.

The relationship between anti-PGL-I seropositivity and the municipality of residence was analyzed as an explanatory variable evaluating the average detection rate of cases per 100,000 inhabitants during the period of 2006–2010. There was a reduction in the seropositivity of 0.99 for every unit increase in the detection rate, but this difference was not significant.

The results of the multivariate analysis are shown in Table 5. The presence of signs suggestive of leprosy (PR = 2.90; *p* = 0.011) and the operational classification (PR = 2.25; *p* = 0.008) were associated with anti-PGL-I seropositivity.

**Table 5:** Final Poisson regression model of the characteristics associated with anti-PGL-I seropositivity among the household contacts

Variable	PR	95 % CI	*P* value
Signs suggestive of leprosys			
No	1		
Yes	2.90	1.26–6.58	0.011
Operational classification			
Paucibacillary	1		
Multibacillary	2.25	1.23–4.11	0.008

*PR* prevalence ratio, *95 % CI* 95 % confidence interval

Hosmer and Lemeshow test: *p* = 1.000

## Discussion

The anti-PGL-I seropositivity (13.5 %) agreed with the range of 10.4 to 28.6 % that has been reported in the literature, including those studies that also applied the ML Flow test to the contacts of leprosy patients [6, 13–18].

The higher prevalence of seropositivity among contacts that showed signs suggestive of leprosy strengthens the rationale of using the ML Flow test to identify individuals at greatest risk of getting sick. The increased risk of developing leprosy in individuals with positive serology has been previously identified in other studies with household contacts [13, 17, 19, 20]. Although none of the contacts referred with suspected leprosy have received confirmation of the diagnosis during the data collection, the largest proportion of contacts with signs suggestive of leprosy among individuals with anti-PGL-I-positive serology suggests that the ML Flow test can also be used for identifying leprosy at an early stage.

Furthermore, a positive result on a serological test to identify antibodies to *Mycobacterium leprae* in individuals with no suggestive signs of disease can be used as a criterion to differentially monitor household contacts, as it is considered a potential indicator of the development of leprosy [21] and because individuals may undergo an extended period of bacillus proliferation before the onset of identifiable clinical signs [22].

Similar results regarding the association between anti-PGL-I seropositivity and the age of the household contact have been observed in other studies [16, 23, 24]. However, there is no consensus in the literature regarding the relationship between anti-PGL-I seropositivity and age.

Considering that children can be exposed to *Mycobacterium leprae* in hyperendemic areas early in life and develop some degree of an immune response via the production of IgM [23], the highest prevalence of positive contacts observed in the 7–14 year age group may be indicative of a high concentration of leprosy cases in the study region.

The anti-PGL-I serology of the contacts may be influenced by the spatial separation between them and the leprosy patients [24, 25]. Moreover, the housing conditions can influence seropositivity, as individuals living with leprosy cases in households with the greatest number of residents and the fewest rooms and, more importantly, the fewest bedrooms, are the most exposed to the bacillus.

The residential variations in spatial distance and the intensity of leprosy exposure may not have reflected differences in the humoral responses of the contacts and by the endemic nature of leprosy in the microregion of Almenara, which might explain our results. Despite a lack of statistically significant differences, we emphasize the importance of assessing the housing conditions in other studies, as both the intensity of leprosy exposure [7, 26] and the housing conditions [23, 27] were associated with an increased risk of developing leprosy.

The anti-PGL-I seropositivity among the contacts of paucibacillary cases (8.4 %) was lower than that reported in other studies, which ranged from 10.8 to 13.5 %. Alternatively, the anti-PGL-I seropositivity among the contacts of multibacillary cases (18.2 %) is within the range of 16.9 to 23.9 % that has been reported in the literature. The lower seropositivity among the contacts of paucibacillary patients than the contacts of multibacillary patients has been observed in other studies [6, 13, 15–17, 22]. That the highest seropositivity prevalence was detected among the contacts of multibacillary cases can be explained by the intensity of their exposure to bacillus because, at this stage, these patients contain a higher bacterial load. This result is also agrees with those of other studies conducted in Brazil [13, 15–17].

The higher prevalence of seropositivity among the contacts of patients with the indeterminate clinical form of leprosy than among the contacts of patients with the tuberculoid form may be related to the potential for indeterminate cases, which do not spontaneously heal, to develop into tuberculoid or lepromatous leprosy. In addition, the anti-PGL-I seropositivity for lepromatous cases tends to be elevated [4].

The relationship observed between the seropositivity of the household contacts and the degree of disability of the index case appears to be reasonable, as the presence of physical disabilities suggests that the diagnosis was late [28] and, therefore, the household contacts of cases with physical disabilities may experience a longer exposure to bacillus. Moreover, the predominance of physical disabilities among the multibacillary leprosy cases that has been described previously [28] suggests a greater intensity of bacillus exposure.

Many studies that have analyzed the anti-PGL-I seropositivity of household contacts have not mentioned certain characteristics of the leprosy index cases, such as the slit skin smear status, the degree of disability at diagnosis and the clinical form of disease [13, 15–17, 23, 29], which limit the comparison of the results.

Other studies of household contacts [30], the general population [31] and schoolchildren [32] in Brazil also found no significant differences between the anti-PGL-I seropositivity and the leprosy detection rate. The observed results can be explained by the influence of operational characteristics related to health services on the detection rate; therefore, these results may not accurately reflect the number of detected cases. In addition, all municipalities studied display a high endemic level of leprosy, and the serological status may not detect small changes in the overall level of endemicity.

Another possible explanation for this observation is that *Mycobacterium leprae* can be transmitted by infected individuals or by bacilli present in the environment or in unknown reservoirs. Furthermore, there exists the possibility of cross-reactivity between anti-PGL-I antibodies and other common infections or environmental mycobacteria [32]. However, the hypothesis of cross-reactivity with other mycobacteria has not been substantiated [24], and studies that have evaluated the cross-reactivity between *Mycobacterium tuberculosis* and other mycobacteria have found no anti-PGL-I seropositivity [33–35].

The association between anti-PGL-I seropositivity and the operational classification of the index leprosy case based on the multivariate analysis was also observed by other authors who performed multivariate analysis [13, 17]. These results can be justified by the higher levels of bacillus exposure among the contacts of multibacillary cases.

The consistent significance of the variables related to signs suggestive of disease in the multivariate analysis model provides further evidence for the use of the ML Flow test to identify individuals at the greatest risk of developing leprosy, especially among the household contacts of leprosy cases [6, 32]. Identifying individuals with early-stage disease and initiating treatment early can help break the chain of transmission. Additionally, we emphasize the importance of the dermatologic evaluation of household contacts of leprosy cases in monitoring the clinical manifestation of the disease, especially among the contacts displaying positive anti-PGL-I serological status, who are more likely to develop leprosy.

## Conclusions

The overall observed anti-PGL-I seropositivity among the household contacts of leprosy cases was 13.5 % and were 8.4 and 17.3 % among contacts of leprosy cases classified as paucibacillary or multibacillary, respectively. The variables associated with the differences in seropositivity in the study population were the presence of signs suggestive of leprosy, the operational leprosy classification and the degree of disability of the index case.

No statistically significant difference was detected between the anti-PGL-I seropositivity and sex, age, education, living conditions, BCG vaccination status, relationship of the contacts or the cohabitation conditions. No significant difference between anti-PGL-I seropositivity and the following characteristics of the leprosy cases was observed: clinical form, slit skin smear status or the number of damaged nerves.

Although no significant difference in anti-PGL-I seropositivity with respect to the housing conditions or the cohabitation characteristics with the index leprosy case was observed, notably, the highest prevalence was observed among the contacts residing in households containing the highest number of residents in the fewest bedrooms and among those who had close contact with a leprosy case. These characteristics are also considered to be risk factors for the development of leprosy. Therefore, it is important to conduct further studies to evaluate these relationships with anti-PGL-I serology.

The serological tests that are available for leprosy are not considered diagnostic tests but can be used as auxiliary assessments in combination with clinical parameters for the classification and clinical monitoring of patients and for identifying exposed individuals who exhibit a high risk of developing leprosy. We emphasize the importance of conducting serological studies, utilizing new markers of infection by *Mycobacterium leprae* and performing longitudinal studies to assess the possibility of the seroconversion of individuals and the evolution or manifestations of leprosy.
